# Delusional Themes Across Affective and Non-Affective Psychoses

**DOI:** 10.3389/fpsyt.2018.00132

**Published:** 2018-04-05

**Authors:** Angelo Picardi, Laura Fonzi, Mauro Pallagrosi, Antonella Gigantesco, Massimo Biondi

**Affiliations:** ^1^Centre of Behavioural Sciences and Mental Health, Italian National Institute of Health, Rome, Italy; ^2^Department of Human Neurosciences, Sapienza University of Rome, Rome, Italy

**Keywords:** delusion, guilt, grandiosity, persecution, mood, psychosis, psychopathology

## Abstract

The current debate about the diagnostic significance of delusion revolves around two positions. The neurocognitive position conceives delusion as a non-specific, though polymorphic, symptom. The psychopathological position views features of delusion such as content and structure as having meaningful connections with diagnostic entities. This study aims at contributing to this debate by examining the association between delusional themes and diagnosis in a sample of 830 adult psychotic patients. All diagnoses were made by experienced psychiatrists according to DSM-IV or ICD-10 criteria, and in 348 patients were established with the SCID-I. All patients were administered the Brief Psychiatric Rating Scale (BPRS). In each patient, the presence of somatic delusions and delusions of guilt, grandiosity, and persecution was determined by examining the scores on relevant BPRS items. Delusions of guilt were almost pathognomonic for a psychotic depressive condition (psychotic major depression 40%; psychotic bipolar depression 30%; depressed schizoaffective disorder 8%; bipolar and schizoaffective mixed states 6 and 7%, respectively). Only 1% of patients with schizophrenia and no patient with delusional disorder or bipolar or schizoaffective manic state showed such delusions. The difference between unipolar and bipolar depression and the other diagnostic groups was highly significant. Delusions of grandiosity characterized mostly patients with manic symptoms (bipolar mania 20%; bipolar mixed states 19%; manic schizoaffective disorder 10%). They were observed significantly more often in bipolar mania than in schizophrenia (7%). Persecutory delusions were broadly distributed across diagnostic categories. However, they were significantly more frequent among patients with schizophrenia and delusional disorder compared with depressed and manic patients. Somatic delusions were also observed in all diagnostic groups, with no group standing out as distinct from the others in terms of an increased prevalence of somatic delusions. Our findings suggest a middle position in the debate between the neurocognitive and the psychopathological approaches. On the one hand, the widespread observation of persecutory delusions suggests the usefulness of searching for non-specific pathogenic mechanisms. On the other hand, the association between some delusional contents and psychiatric diagnosis suggests that a phenomenological analysis of the delusional experience may be a helpful tool for the clinician in the diagnostic process.

## Introduction

Since the beginning of the discipline, delusion has attracted immense interest in the field of psychiatry and has been viewed as a basic, distinctive feature of mental illness. Over the centuries, it has been interpreted in various ways and has been given different meanings, especially with regard to its diagnostic significance. Some types of delusions have been considered pathognomonic of certain conditions, such as delusions of being controlled and of thought withdrawal, insertion, and broadcasting for schizophrenia (1), and delusions of guilt and hypochondriacal delusions in melancholia (2). A careful examination of delusional contents and of their relationship with the underlying disorder was also seen as important for differential diagnosis (2–5).

More recently, and especially during the last two decades, the relationship between delusional themes and diagnosis has lost its centrality and has remained on the sidelines of the nosological debate, to the extent that the last DSM criterion making reference to a typical aspect of delusional experience, i.e., bizarre delusions for schizophrenia, was removed from the latest edition of the manual (6).

Within the current lively debate between leading researchers about how to best conceptualize and study delusions, two major approaches can be distinguished (7). On the one hand, the scholars from the neurocognitive approach conceive and investigate delusions as separate transdiagnostic phenomena, sharing a similar pattern of formation and evolution, attributable to specific cognitive mechanisms and possibly to corresponding neurobiological pathways (8–10). This approach, which stems mainly from studies of persecutory delusions (11), considers delusions dimensionally along a continuum from normality to severe pathology (12–14) and focuses on a number of cognitive and affective styles, life events, and early childhood experiences (9, 10, 15–17). In particular, the model that has received the most empirical support (9) involves two main categories of cognitive bias: the “jumping to conclusions” and the “evidence integration” biases. The first is the tendency to make rushed and premature decisions on the basis of little evidence. The second involves an impairment in the integration of disambiguating evidence, which may result from biases against confirmatory or disconfirmatory evidence and from a “liberal acceptance” bias characterized by proneness to accept implausible interpretations (18). According to this model (11), in persons with such cognitive biases and a proneness to negative emotions and cognitions (e.g., low self-esteem, self-critical thinking), some internal or external events may trigger a search for meaning, which can lead to delusional beliefs. Then, the same cognitive biases involved in the development of delusional beliefs may contribute to maintain and expand such beliefs. While this model is based on studies of persecutory delusions across different diagnostic categories, some attempts have been made to extend it to the genesis of grandiose delusions (16, 17). Overall, the main aim of this approach is the identification of specific targets for therapeutic interventions for delusional thoughts, regardless of specific diagnosis (10, 19).

On the other hand, the scholars who follow the classical psychopathological approach (7, 20–22) hold the view that only a rigorous phenomenological examination of the delusional experience within the context of the whole personality allows to distinguish between different types of delusion. The delusional experience is seen as strongly linked to the overall psychopathological experience, with which it shares the basic structures, such as the relationship with the self, the intersubjective dimension, the spatial and temporal aspects, and the nature of its contents (7, 20, 21). In other words, features of delusion and diagnostic entities cannot be separated without losing meaningful connections. For instance, the phenomenological perspective makes a clear distinction between schizophrenic and melancholic delusions. On the one hand, the bizarre nature of the former is considered to be expression of a deep fragmentation of self-experience (23); on the other hand, the latter are believed to originate from the deadly experience of depressive guilt (22), even when it reaches the level of a temporary psychotic break from reality.

Such a debate involves different epistemologies (20) and has important diagnostic and therapeutic implications. It is an important question whether the careful analysis of delusions in terms of content, structure, and relationship with other symptoms such as disordered mood (24) and hallucinations (25) may contribute to a more accurate diagnostic evaluation, or whether it can be dismissed in favor of a view of delusion as a non-specific, though polymorphic, symptom needing focused attention and treatment. Until now, the empirical literature does not seem to provide a definite answer to this question, as the available evidence does not allow to firmly reject any of the two hypotheses. Only few studies have specifically investigated the distribution of different types of delusion across major psychiatric disorders. Also, they were carried out on different mix of diagnoses, such as all psychotic disorders, or only some of these disorders, or psychotic and substance use disorders together.

The majority of the studies that examined the prevalence of different kinds of delusions in heterogeneous samples of patients with psychotic symptoms have reported consistent prevalence estimates (13–15, 17, 26–30). The most prevalent delusions, though often coexisting with other types of delusions, are those of persecution, reference, grandiosity, thought control, insertion, withdrawal, and broadcasting. A lower frequency has been reported for other types of delusions, such as delusions of guilt, somatic delusions, and delusions of infidelity.

Only some of these studies have examined the distribution of delusional themes in different diagnostic groups. A number of studies have specifically focused on the differential diagnosis between schizophrenia spectrum disorders and either delusional disorder (14, 31) or bipolar disorder (14, 26, 29, 32). These studies reported a greater prevalence of delusions of thought control, insertion, withdrawal, and broadcasting in patients with schizophrenia (14, 26) and of delusions of grandiosity in patients with bipolar disorder experiencing a manic episode (26, 29). The latter finding has been corroborated by further studies, which showed that delusions of grandiosity are only typical of manic states, whereas the overall course of the disorder is mostly characterized by persecutory delusions (33). Interestingly, a recent study on patients with first-episode psychosis reported that the presence of grandiose delusions with religious content was associated with a diagnostic shift from non-affective psychosis to bipolar disorder on the second admission (32).

Only a few studies have investigated the delusional themes that are classically associated with psychotic depression. As far as somatic delusions are concerned, these studies did not report significant differences in frequency between depression and other major psychiatric disorders (14). Moreover, somatic delusions have also been observed in studies that did not include depressed patients (13, 17, 29). Concerning delusions of guilt, the findings are less clear. On the one hand, such delusions have been observed only in a very small proportion of non-depressed psychotic patients (13, 15, 17, 29, 31). On the other hand, the only two studies that directly compared depressed and non-depressed psychotic patients yielded contrasting results. Whereas one study reported that delusions of guilt significantly discriminated between patients with psychotic depression and patients with schizophrenia (27), the other reported that such delusions did not differentiate between depression and schizophrenia spectrum disorders (14).

This study aims at contributing to the debate on the complex relationship between types of delusion and diagnosis by examining the association between a number of delusional themes and major psychiatric disorders in a large sample of psychotic patients. We examined the frequency of somatic delusions, persecutory delusions, delusions of grandiosity, and delusions of guilt among psychotic patients with a diagnosis of schizophrenia, delusional disorder, schizoaffective disorder, bipolar disorder, and major depression. To best determine the relationship between mood and delusions of grandiosity and guilt, we took into account the current polarity of mood both for patients with bipolar disorder and patients with schizoaffective disorder. Based on the available clinical and research literature, we hypothesized that grandiose delusions and delusions of guilt would be associated with manic and depressive states, respectively, and that persecutory and somatic delusions would not show an association with specific diagnoses.

## Materials and Methods

### Participants

The patients included in this study come from a recently completed study performed at the psychiatric department of the Umberto I Policlinico Hospital, Sapienza University of Rome, and from three previous studies carried out in a sample of acute inpatient units spread across Italy (34, 35), in a number of psychiatric inpatient and outpatient units in Rome (36), and in several community mental health centers distributed across Italy (37). A total of 830 adult patients aged 18–65, who were free from intellectual disability or severe cognitive impairment and were diagnosed with a major psychotic disorder (schizophrenia, paranoid, disorganized, catatonic, or undifferentiated type; delusional disorder; schizoaffective disorder; bipolar I disorder, current episode manic, or mixed with psychotic features; bipolar I or II disorder, current episode depressed with psychotic features; major depressive disorder with psychotic features) were included in the study. Table 1 summarizes the clinical and demographic characteristics of all study participants. According to the Italian legislation, this study does not need formal ethical approval because it is a retrospective, purely observational study based on data collected as part of routine patient assessment.

**Table 1:** Patient characteristics.

	Schizophrenia (*N* = 318)		Delusional disorder (*N* = 95)		Schizoaffective disorder (*N* = 118)		Bipolar disorder (*N* = 217)		Major depressive disorder (*N* = 82)	
					
	*N* (%)	Mean ± SD	*N* (%)	Mean ± SD	*N* (%)	Mean ± SD	*N* (%)	Mean ± SD	*N* (%)	Mean ± SD
**Sex**										
Male	213 (67.0)		41 (43.2)		58 (49.2)		106 (48.8)		29 (35.4)	
Female	105 (33.0)		54 (56.8)		60 (50.8)		111 (51.2)		54 (64.6)	
**Age**		39.1 ± 11.5		53.9 ± 10.8		40.6 ± 10.7		39.7 ± 12.7		44.1 ± 12.3

**Education**										
Total years of education		10.4 ± 3.5		10.5 ± 4.2		10.8 ± 3.7		11.4 ± 6.3		10.1 ± 3.7
Primary school	35 (11.5)		16 (17.6)		14 (12.1)		20 (9.6)		16 (20.0)	
Junior high school	126 (41.3)		34 (37.4)		41 (35.3)		61 (29.3)		25 (31.2)	
Senior high school	118 (38.7)		23 (25.3)		46 (39.7)		96 (46.2)		33 (41.2)	
University degree	26 (8.5)		18 (19.8)		15 (12.9)		31 (14.9)		6 (7.5)	

**Marital status**										
Unmarried	250 (79.1)		49 (51.6)		81 (69.2)		123 (56.7)		24 (29.3)	
Married	30 (9.5)		31 (32.6)		19 (16.2)		63 (29.0)		41 (50.0)	
Separated or divorced	31 (9.8)		10 (10.5)		15 (12.8)		29 (13.4)		16 (19.5)	
Widowed	5 (1.6)		5 (5.3)		2 (1.7)		2 (0.9)		1 (1.2)	

**Diagnosis**										
Schizophrenia, paranoid type	235 (73.9)									
Schizophrenia, disorganized type	53 (16.7)									
Schizophrenia, catatonic type	5 (1.6)									
Schizophrenia, undifferentiated type	25 (7.9)									
Delusional disorder			95 (100)							
Schizoaffective disorder, manic type					29 (24.6)					
Schizoaffective disorder, mixed type					29 (24.6)					
Schizoaffective disorder, depressive type					60 (50.8)					
Bipolar I disorder, current episode manic							162 (74.7)			
Bipolar I disorder, current episode mixed							32 (14.7)			
Bipolar disorder, current episode depressed, with psychotic features							23 (10.6)			
Major depressive disorder, with psychotic features									82 (100)	
**SCID-based diagnosis**	116 (36.5)		42 (44.2)		40 (33.9)		99 (45.6)		51 (62.2)	
**Brief Psychiatric Rating Scale total score**		60.3 ± 16.4		54.8 ± 14.8		59.1 ± 15.5		57.3 ± 18.2		58.4 ± 11.6

*Numbers may not add to total and 100% in each group, due to a few missing data*.

### Procedure

In both the recently completed study and the study carried out in community mental health centers across Italy (37), psychiatric diagnoses were established by trained clinicians using the Structured Clinical Interview for DSM-IV-TR Axis I Disorders, Research Version, Patient Edition (SCID-I) (38). It is a clinician-administered interview that covers most Axis I disorders and is regarded as the standard for making DSM-IV diagnoses. Several studies showed its superior validity over standard clinical interviews at intake episode (39, 40). A total of 348 patients were administered the SCID-I. In the other studies (34–36), involving a total of 482 patients, the diagnoses were made by experienced psychiatrists according to DSM-IV or ICD-10 criteria.

In all studies, the patients were administered the Brief Psychiatric Rating Scale (BPRS) (41, 42), which is a clinician-rated instrument consisting of 24 items, scored on a 7-point severity scale. We used a validated Italian version, which has high reliability (43) and is based on the BPRS manual of administration, with defined anchor points and detailed probe questions and rules for scoring (42). Higher scores indicate greater severity of psychiatric symptoms.

All clinicians received specific training in administering and scoring the BPRS. Also, all clinicians who administered the SCID-I were trained in the use of the interview. They based their assessment on all sources of information available, including not only patients’ answers and clinical observation, but also referral notes, medical records, and reports by significant others. The inter-rater reliability was assessed on several occasions using videotaped interviews. A total of 12 interviews of patients representing all the main diagnostic entities were used for reliability assessments. The interviews were presented to groups of raters, then each rater independently completed the assessment instruments, and subsequently either weighted kappa for multiple raters or intraclass correlation coefficient, as appropriate, was computed to measure reliability. Overall, the reliability of the SCID-I as measured by the kappa coefficient was satisfactory; it ranged from 0.75 to 0.94 for the most common diagnoses (psychotic, mood, anxiety, and eating disorders). The mean inter-rater reliability of the BPRS as measured by the intraclass correlation coefficient was also satisfactory, since it ranged from 0.81 to 0.93.

### Statistical Analysis

All statistical analyses were performed using SPSS Statistics for Mac, Version 20 (IBM Corp., Armonk, NY, USA). All statistical tests were two-tailed, with alpha set at 0.05. First, patient characteristics were summarized using appropriate descriptive statistics. Then, four dichotomous variables were created using the cut-off score of 6 on the BPRS “Somatic Concern,” “Guilt,” “Grandiosity,” and “Suspiciousness” items. For grandiose and persecutory delusions, a score of 6 or more on the relevant BPRS item is sufficient to infer the presence of a delusion, as the scoring instructions for item 8 “Grandiosity” and item 9 “Suspiciousness” explicitly state that a patient who receives a rating of 6 is delusional. For somatic delusions and delusions of guilt, such a score alone is not sufficient to infer the presence of a delusion, as the scoring instructions for items 1 “Somatic concern” and item 5 “Guilt” state that the patient who receives a rating of 6 may either be delusional or have “preoccupation with somatic complaints with much impairment in functioning” or “unreasonable self-reproach very out of proportion to circumstances,” respectively. However, in combination with a score of 4 (“delusion present but no preoccupation or functional impairment”) or more on item 11 “Unusual Thought Content,” such very high scores on these four BPRS items suggest the presence of somatic delusions, delusions of guilt, delusions of grandiosity, and persecutory delusions, respectively. We categorized patients as having these types of delusions or not based on the combination of scores on the five BPRS items mentioned above.

Then, the Chi square test was used to test for differences between groups in categorical variables. To investigate further statistically significant omnibus chi-square test results and evaluate whether specific cells differ from each other, we followed the procedure described by Goodman (44, 45). In short, a *z*-test was calculated as follows: *z* (Ψ−0)/SEψ with Ψ being the contrast of interest and SEψ being the standard error of that contrast, Then, the *z* obtained value was conservatively tested, not merely against the *z* critical value for alpha = 0.05, but rather against square root of the chi-square critical value for the entire contingency table (46). Given the fairly large number of cells in each contingency table, all *z*-tests were adjusted using a Bonferroni correction. Analysis of variance was used to test for differences between groups in continuous variables. Tukey’s *post hoc* test was used for pairwise comparisons between groups.

## Results

There were a few significant differences in demographic variables between groups. Several of these differences were small in magnitude and were due to the high statistical power afforded by the large sample size. Patients with schizophrenia (*p* < 0.01) and bipolar disorder (*p* < 0.05) were significantly younger than patients with major depression and delusional disorder. Patients with major depression were significantly more likely than patients in all other groups to be married (*p* < 0.001), while patients with bipolar disorder and delusional disorder were significantly more likely to be married than patients with schizophrenia (*p* < 0.001). As far as education is concerned, patients with bipolar disorder had significantly more years of education than patients with schizophrenia (*p* < 0.05). In terms of degrees, patients with delusional disorder were less likely than patients with bipolar disorder to have completed senior high school (*p* < 0.05) but were more likely than patients with schizophrenia to have a university degree (*p* < 0.05). Finally, the patients with schizophrenia had significantly higher scores on the BPRS than the patients with delusional disorder (*p* < 0.05).

The overall prevalence of delusions in our sample, as indicated by a score of 4 or more on the BPRS Unusual Thought Content item, alone or in combination with a score of 6 or more on the BPRS Somatic Preoccupation, Guilt, Grandiosity, and Suspiciousness items, was 74.1%.

The presence of delusions of guilt was significantly associated with higher age (*p* < 0.05), married vs. unmarried marital status, and higher total BPRS score (*p* < 0.01), whereas it was not associated with sex or education. Delusions of grandiosity were associated with male sex (*p* < 0.01), better education (*p* < 0.05), and higher total BPRS score (*p* < 0.001), while they were not associated with age and marital status. Persecutory delusions were associated with higher total BPRS score (*p* < 0.001), while they showed no association with age, sex, marital status, and education. Somatic delusions were associated with better education (*p* < 0.05) and higher total BPRS score (*p* < 0.001), whereas they displayed no association with age, sex, and marital status.

**Figure 1:**
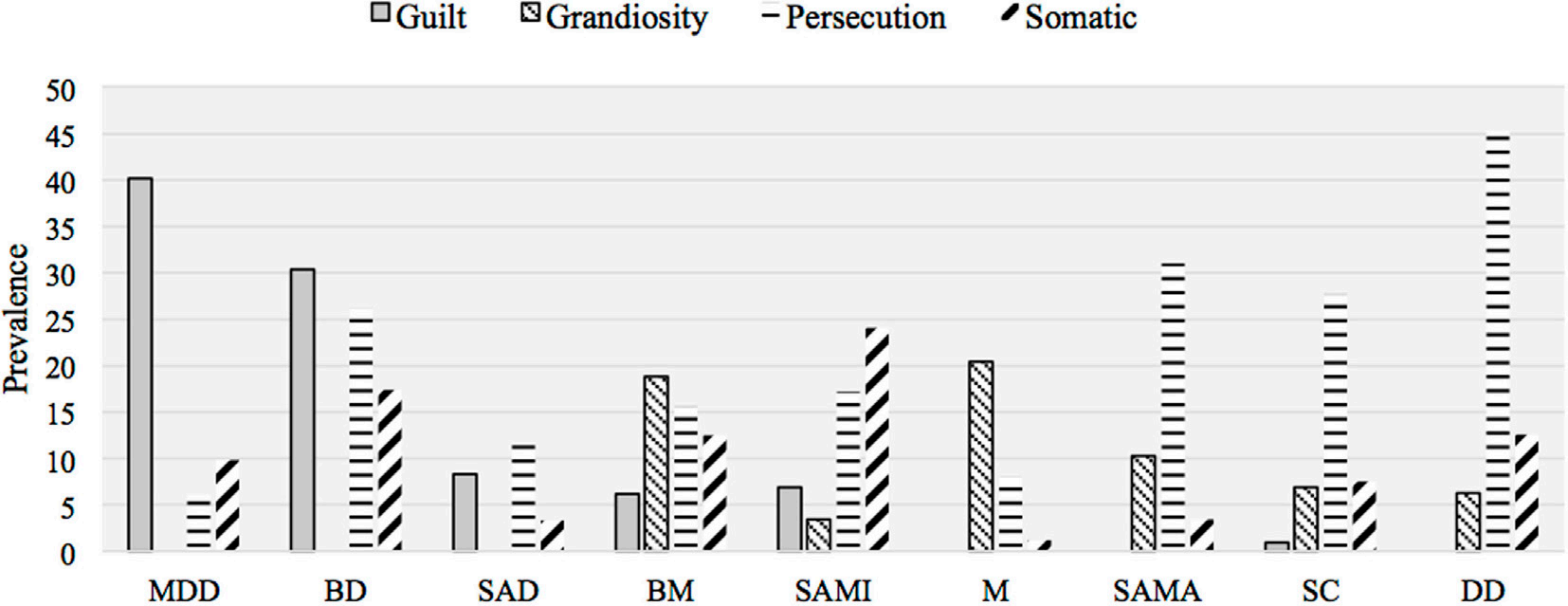
Prevalence of delusional themes by diagnostic group (*N* = 830). MDD, major depressive disorder; BD; bipolar disorder, depressive episode; SAD, schizoaffective disorder, depressive type; BM, bipolar disorder, mixed episode; SAMI, schizoaffective disorder, mixed type; M, mania; SAMA, schizoaffective disorder, manic type; SC, schizophrenia; DD, delusional disorder.

Table 2 and Figure 1 illustrate the distribution of specific delusional themes by group. The standardized residuals for each cell, which express the strength of the difference between observed and expected values, are reported in Table S1 in Supplementary Material. Delusions of guilt were almost pathognomonic for a psychotic depressive condition, as they were found in 40% of patients with psychotic major depression, 30% of those with psychotic bipolar depression, 8% of those with schizoaffective disorder, depressed type, and 6 and 7% of patients with bipolar and schizoaffective mixed states, respectively. On the contrary, only 1% of patients with schizophrenia and no patient with either delusional disorder or bipolar or schizoaffective manic state showed delusions of this kind. The overall difference between diagnostic groups in the proportion of patients with delusions of guilt was significant (*p* < 0.001). In *post hoc* analysis, the patients with psychotic major depression significantly differed from all other diagnostic groups (*p* < 0.001) except those with psychotic bipolar depression, which in turn significantly differed from all the remaining groups (*p* < 0.001) except for patients with bipolar mixed states and depressive or mixed schizoaffective disorder.

**Table 2:** Presence of specific delusional themes by diagnostic group.

	Delusions of guilt	Delusions of grandiosity	Persecutory delusions	Somatic delusions	None of these delusional types
Schizophrenia	3 (0.9%)	22 (6.9%)	88 (27.7%)	24 (7.5%)	209 (65.7%)
Delusional disorder	0	6 (6.3%)	43 (45.3%)	12 (12.6%)	44 (46.3%)
Schizoaffective disorder, manic type	0	3 (10.3)	9 (31.0%)	1 (3.4%)	18 (62.1%)
Schizoaffective disorder, mixed type	2 (6.9%)	1 (3.4%)	5 (17.2%)	7 (24.1%)	18 (62.1%)
Schizoaffective disorder, depressive type	5 (8.3%)	0	7 (11.7%)	2 (3.3%)	47 (78.3%)
Bipolar I disorder, current episode manic	0	33 (20.4)	13 (8.0%)	2 (1.2%)	123 (75.9%)
Bipolar I disorder, current episode mixed	2 (6.2%)	6 (18.8%)	5 (15.6%)	4 (12.5%)	22 (68.8%)
Bipolar disorder, current episode depressed with psychotic features	7 (30.4%)	0	6 (26.1%)	4 (17.4%)	11 (47.8%)
Major depressive disorder with psychotic features	33 (40.2%)	0	5 (6.1%)	8 (9.8%)	40 (48.8%)
Between-group differences	MDD > all other groups except BD (*p* < 0.001) BD > all other groups except BM, SAMI, and SAD (*p* < 0.001)	M > MDD, SAD, SC (*p* < 0.001) BM > MDD, SAD (*p* < 0.001)	DD > MDD, M, SAD (*p* < 0.001), SC (*p* < 0.05) SC > MDD and M (*p* < 0.001)	BD, DD, BM, and SAMI > M (*p* < 0.05)	

*MDD, major depressive disorder; BD; bipolar disorder, depressive episode; BM, bipolar disorder, mixed episode; SAMI, schizoaffective disorder, mixed type; SAD, schizoaffective disorder, depressive type; M, mania; SC, schizophrenia; DD, delusional disorder*.

Delusions of grandiosity characterized mostly patients with mania (20%) and bipolar mixed states (19%), as well as those with manic schizoaffective disorder (10%). However, they were also observed in a non-negligible proportion of patients with schizophrenia (7%), delusional disorder (6%), and mixed schizoaffective disorder (3%). As expected, no patient with a depressive condition showed such delusions. The overall difference between diagnostic groups in the proportion of patients with delusions of grandiosity was significant (*p* < 0.001). In *post hoc* analysis, patients with a bipolar manic (*p* < 0.001) or mixed (*p* < 0.05) state significantly differed from those with psychotic major depression and depressive schizoaffective disorder; also, manic patients significantly differed from patients with schizophrenia (*p* < 0.001).

Persecutory delusions were observed in all diagnostic groups, although to different degrees. Such delusions distinguished mostly patients with delusional disorder (45%) but were also frequent in patients with schizophrenia (28%), manic schizoaffective disorder (31%), and psychotic bipolar depression (26%). A lower prevalence of delusions of this kind was found in psychotic major depression (6%), mania (8%), and mixed (17%), and depressed (12%) schizoaffective disorder. The overall difference between diagnostic groups in the proportion of patients with delusions of grandiosity was significant (*p* < 0.001). In *post hoc* analysis, psychotic major depression and mania were significantly different from schizophrenia and delusional disorder (*p* < 0.001). Also, delusional disorder was significantly different from depressive schizoaffective disorder (*p* < 0.001) and schizophrenia (*p* < 0.05).

Somatic delusions were observed in a sizable proportion in patients with psychotic unipolar (10%) and bipolar (17%) depression, bipolar mixed states (13%), schizophrenia (8%), delusional disorder (13%), and mixed schizoaffective disorder (24%). They were rarely observed in manic patients (1%) and in patients with manic or depressive schizoaffective disorder (both 3%). The overall difference between diagnostic groups in the proportion of patients with somatic delusions was significant (*p* < 0.001). In *post hoc* analysis, mania significantly differed (*p* < 0.05) from psychotic bipolar depression, delusional disorder, bipolar mixed states, and mixed schizoaffective disorder.

The proportion of patients with a given delusional theme who did not show any of the other three themes studied was 77% for patients with delusions of guilt, 52% for patients with grandiose delusions, 70% for patients with persecutory delusions, and 48% for patients with somatic delusions.

We performed a series of additional analyses to investigate the possible confounding effect of diagnostic assessment methodology, demographic variables, and overall clinical severity. To this purpose, we repeated the analysis after stratifying for SCID-based vs. clinical diagnosis, median age, sex, median years of education, marital status (married vs. others), and median BPRS score. In each of these analyses, the findings were similar to those observed in the full sample.

Table 3 illustrates the distribution of specific delusional themes by group in patients with SCID-based diagnoses only. The Chi square test was significant for all delusional themes (*p* < 0.001 for delusions of guilt, grandiosity, and persecution; *p* < 0.05 for somatic delusions). The standardized residuals for each cell are reported in Table S2 in Supplementary Material. Delusions of guilt were still quite specific for a psychotic depressive condition, as they were observed in 47% of patients with psychotic major depression, 43% of those with psychotic bipolar depression, 8% of those with depressive schizoaffective disorder, and 9% of patients with bipolar mixed states. On the contrary, only 1.7% of patients with schizophrenia, and no patient with delusional disorder, mania, and manic or mixed schizoaffective disorder displayed delusions of this kind. The analysis still indicated a significant (*p* < 0.001) difference between patients with psychotic major depression and all other diagnostic groups except those with psychotic bipolar depression and bipolar mixed states. In turn, the patients with bipolar depression significantly differed from all the remaining groups (*p* < 0.01) except patients with bipolar mixed states and depressive schizoaffective disorder.

**Table 3:** Presence of specific delusional themes by diagnostic group in patients diagnosed with the SCID-I (*N* = 348).

	Delusions of guilt	Delusions of grandiosity	Persecutory delusions	Somatic delusions	None of these delusional types
Schizophrenia	2 (1.7%)	7 (6.0%)	33 (28.4%)	7 (6.0%)	76 (75.5%)
Delusional disorder	0	1 (2.4%)	21 (50.0%)	3 (7.1%)	19 (45.2%)
Schizoaffective disorder, manic type	0	2 (22.2)	1 (11.1%)	0	6 (66.7%)
Schizoaffective disorder, mixed type	0	0	1 (14.3%)	1 (14.3%)	5 (71.4%)
Schizoaffective disorder, depressive type	2 (8.3%)	0	4 (16.7%)	0	18 (75.0%)
Bipolar I disorder, current episode manic	0	16 (21.6)	7 (9.5%)	1 (1.4%)	54 (73.0%)
Bipolar I disorder, current episode mixed	1 (9.1%)	1 (9.1%)	3 (27.3%)	1 (9.1%)	7 (63.6%)
Bipolar disorder, current episode depressed with psychotic features	6 (42.9%)	0	6 (42.9%)	4 (28.6%)	3 (21.4%)
Major depressive disorder with psychotic features	24 (47.1%)	0	3 (5.9%)	6 (11.8%)	22 (43.1%)
Between-group differences	MDD > all other groups except BD and BM (*p* < 0.001) BD > all other groups except BM and SAD (*p* < 0.01)	M > MDD and SC (*p* < 0.001) SAMA > MDD (*p* < 0.05)	SC (*p* < 0.05), DD (*p* < 0.01), and BD (*p* < 0.05) > MDD DD (*p* < 0.01) and BD (*p* < 0.05) > M	BD > M (*p* < 0.01)	

*MDD, major depressive disorder; BD; bipolar disorder, depressive episode; BM, bipolar disorder, mixed episode; SAD, schizoaffective disorder, depressive type; M, mania; SAMA, schizoaffective disorder, manic type; SC, schizophrenia; DD, delusional disorder*.

Delusions of grandiosity still characterized mostly patients with mania and manic schizoaffective disorder (both 22%), as well as those with bipolar mixed states (9%). However, they were also observed in a non-negligible proportion of patients with schizophrenia (6%) and delusional disorder (2%). The analysis still indicated that patients with mania significantly differed (*p* < 0.001) from those with psychotic major depression and schizophrenia. Also, the patients with manic schizoaffective disorder significantly differed from those with psychotic major depression (*p* < 0.05).

Persecutory delusions were still observed in all diagnostic groups, although to different degrees, and were found to distinguish patients with delusional disorder (50%), but they were present in all other patient groups. They were frequent in patients with schizophrenia (28%), bipolar mixed states (27%), and psychotic bipolar depression (43%) and were also observed in psychotic major depression (6%), mania (9%), and manic (11%), mixed (14%), and depressive (17%) schizoaffective disorder. The analysis still showed that psychotic major depression was significantly different from schizophrenia (*p* < 0.05) and delusional disorder (*p* < 0.01), as well as from bipolar depression (*p* < 0.05). Also, delusional disorder (*p* < 0.01) and bipolar depression (*p* < 0.05) were significantly different from mania.

Somatic delusions were still observed in a fair proportion in patients with psychotic unipolar (12%) and bipolar (29%) depression, bipolar mixed states (9%), schizophrenia (6%), delusional disorder (7%), and mixed schizoaffective disorder (14%). They were rarely observed in manic patients (1%) and were not found in patients with manic or depressive schizoaffective disorder. The analysis indicated that mania significantly differed (*p* < 0.05) from psychotic bipolar depression.

## Discussion

The study sample seems representative of patients with psychotic conditions encountered in everyday clinical practice. Most of the differences observed between diagnostic groups in demographic variables were small in magnitude and should not be over-interpreted as they were mainly due to the high statistical power afforded by the large sample size. In any case, these differences are consistent with the known demographic characteristics of the disorders studied. The patients with major depression were older as compared with the patients affected by disorders with onset in early adulthood, such as schizophrenia and bipolar disorder, and were more likely to be married. The patients with delusional disorder, which typically appears in middle age, were older than the patients with schizophrenia and bipolar disorder, and were more educated than the former and less educated than the latter.

The correlations observed between the different types of delusion, demographic variables, and overall clinical severity are also plausible. The associations of delusions of guilt with age and marital status are explained by the strong association between this delusion type and major depression. Also, as expected, all four types of delusion were associated with greater overall clinical severity as measured by the BPRS.

Far more important, from a clinical perspective, are the findings regarding the distribution of delusion types across the different diagnostic categories. Somatic delusions were present in all diagnostic groups, though they were only rarely observed in manic and depressed schizoaffective patients, and especially in manic bipolar patients. Indeed, the proportion of patients with bipolar mania that presented somatic delusions was significantly lower as compared with the patients affected by delusional disorder, depressed or mixed bipolar disorder, and mixed schizoaffective disorder. No diagnostic group stood out as distinct from the others in terms of an increased prevalence of somatic delusions. Previous studies of psychotic patients did not report a significant association between somatic delusions and diagnosis (14, 27, 29), with the exception of a study that found a greater frequency of such delusions among patients with delusional disorder compared with patients with schizophrenia (31). This apparent lack of specificity seems plausible, given the polymorphic nature of somatic delusions, which can vary from monothematic forms more typical of paranoia (e.g., delusions of parasitosis) to delusional perceptions regarding body parts as in some schizophrenic delusions, to delusions of being dead or rotting as in severe melancholia. Possibly, only a more in-depth analysis of the content of each somatic delusion may allow identifying patterns of presentation typical for different disorders, as a previous study seems to suggest (47).

As previously reported in the literature, in our patients, persecutory delusions were broadly distributed across all diagnostic categories, although they were significantly more frequent among patients with schizophrenia and delusional disorder compared with depressed and manic patients. The relatively common observation of delusions with themes other than grandiosity, guilt, death, or nihilism in patients with a diagnosis of mood disorder lies at the heart of the long-standing, and still not fully resolved, controversy in the literature about the classification of mood disordered patients with mood-incongruent psychotic symptoms, with opinions ranging from conceptualizing such patients as typical cases of affective illness to viewing them as suffering from a form of schizophrenia (48). From another perspective, the observation of persecutory delusions across a wide range of psychotic disorders with putatively different pathogenesis is the foundation on which the research on the common mechanisms of formation of paranoid delusions is based (11, 15, 49, 50). This research has subsequently expanded beyond a focus on persecutory delusion to encompass the cognitive and emotional mechanisms underlying delusion in general (10, 19).

Delusions of grandiosity were also present in all diagnostic groups except depressed patients, either unipolar, bipolar, or schizoaffective. However, such delusions were observed much more often in manic and mixed bipolar patients. The proportion of patients with bipolar mania that showed grandiose delusions was significantly higher compared with patients with schizophrenia and the various depressive conditions. Previous studies yielded similar results regarding the scarcity of grandiose delusions among depressed patients (14) and the greater prominence of such delusions among manic patients as compared with schizophrenic patients (14, 26, 29, 51). A recent review of the phenomenology of psychotic symptoms in patients with bipolar disorder has also suggested that delusions of grandiosity are the most prevalent kind of delusions during manic episodes (33). Overall, our findings, together with those of previous studies, suggest a close link between mood and the most prominent delusional theme, which actually once again raises the issue of the differential diagnosis between affective and non-affective psychosis. It is no coincidence that several authors have attempted to delineate similarities and differences between persecutory and grandiose delusions, albeit reaching different conclusions (16, 17, 52). While the issue is still open, both our findings and previous reports suggest that although grandiose delusions can be observed in non-affective psychoses, the presence of such delusions should prompt the clinician to reflect carefully about the possible affective nature of a psychotic illness (32).

Of note, our finding that only about one fifth of manic patients displayed grandiose delusions suggests that, while these delusions are quite characteristic of mania, the manic state is more often characterized by other psychotic symptoms, such as hallucinations, disorganized behavior or speech, and abnormal motor behavior. In our patients with mania, the prevalence of any other type of delusion was 29.6% and the overall proportion of delusional patients was 45.7%. Hints about the prevalence of severe levels of other important, non-delusional symptoms apart from elevated mood are provided by the observation of high (≥5) scores on the relevant BPRS items. Such high scores were found in 9.9% of manic patients for hallucinations and in 9.3, 17.9, 10.5, 38.3, and 24.7% of patients for hostility, bizarre behavior, conceptual disorganization, excitement, and motor hyperactivity, respectively. Indeed, Kraepelin himself, in his description of delusional mania (53), seemed to suggest that only a portion of manic patients experience clear-cut delusions: “The delusions and hallucinations, which in the morbid states hitherto described are fugitive or merely indicated, acquire in a series of cases an elaboration which calls to mind paranoid attacks.” Delusions were also not identified in a non-negligible portion of patients with diagnoses other than mania. When the whole sample is considered, about one fourth of patients (25.9%) did not display any type of delusion. This finding is consistent with the non-delusional nature of many of the qualifying symptoms for psychotic disorders.

The type of delusion showing the strongest association with diagnosis was delusion of guilt, to the extent that it emerged as almost pathognomonic of psychotic depressive conditions. Its prevalence in major depression was significantly higher than in any other diagnostic group, with the exception of bipolar depression that, in turn, showed a significantly higher prevalence than all the other groups except those characterized by some amount of depressive symptoms (i.e., depressive schizoaffective and mixed bipolar or schizoaffective patients). Moreover, this type of delusion was not observed in any patient with a bipolar or schizoaffective manic mood state, in any patient with delusional disorder, and only in a very small proportion of patients with schizophrenia. Consistent with our study, previous studies reported that delusion of guilt was rarely observed in non-depressed psychotic patients (13, 15, 17, 29, 31) and that it significantly discriminated between patients with psychotic depression and patients with schizophrenia (27). However, in these studies, some patients with non-depressive psychotic conditions showed delusion of guilt, and in another study, this type of delusion did not differentiate between depression and schizophrenia spectrum disorders (14). A possible explanation for the discrepancy in findings between this study and previous work is that in all previous studies the schizophrenia spectrum disorder group included also the patients with schizoaffective disorder. Given that our study and previous research (54) suggest that delusion of guilt is relatively common among non-manic schizoaffective patients, their inclusion within the schizophrenia spectrum disorder group may have inflated the prevalence of delusions of guilt in this group in previous studies.

The discriminant value of delusion of guilt for the diagnosis of depression does not support the hypothesis that all delusions result from similar pathogenic mechanisms. As far as the differentiation between affective and non-affective psychoses is concerned, this finding is compatible with the hypothesis proposed by classical psychopathologists that the phenomenological analysis of the backbone of delusional experience may allow to better distinguish between different psychotic disorders (22). Our findings concerning grandiose delusions are also compatible with this hypothesis, though with greater caution given that these delusions were also observed in a small, but non-negligible, proportion of patients with non-affective psychoses. However, given some limitations as described below, this study does not allow drawing firm conclusions about this issue, which could be better elucidated by future studies investigating a broader range of delusional themes together with affective symptoms and emotional and cognitive processes.

This study has some limitations. First, although in principle the BPRS allows a reliable and standardized assessment of the presence of the delusion types that we investigated, there is a limit to the degree to which this can be achieved in practice, as the BPRS is not specifically designed to assess delusions. Second, the BPRS does not allow to perform a sophisticated assessment of delusion content, pervasiveness, development, and evolution. For instance, as previously touched upon, it has not been possible to figure out the exact nature of the delusional content of a given somatic delusion that can range between two poles with profoundly different meanings, i.e., hypochondriacal delusions and delusions of bodily change. A third, related limitation is that it has not been possible to study all delusional themes, but only the four themes specifically assessed by the BPRS. Although the delusional themes that were examined are among the most often encountered in clinical practice, some important delusion types, such as delusions of reference, infidelity, ruin, thought control, insertion, withdrawal, and broadcasting, could not be investigated. Fourth, in about half of the patients, the diagnosis was not established using a standardized clinical interview. However, all diagnoses were made according to ICD or DSM criteria, and identical findings were observed when only patients diagnosed with the SCID-I were included in the analysis. Fifth, the cross-sectional design makes it impossible to exclude that some patients may have previously or subsequently shown a delusional theme that they were not displaying when the evaluation took place. Therefore, the interpretation of the findings is limited to the current psychotic episode and cannot be generalized to the entire course of the disorders examined in this study. Finally, the assessment of the main study variables was not independent, as the same psychiatrist who completed the BPRS was also frequently involved in making the diagnosis. This carries the risk of circularity bias, as the presence of a specific kind of delusion (e.g., guilt) may have increased the likelihood of making a specific diagnosis (e.g., psychotic depression). This risk is an inherent problem for studies of this kind, and it is particularly high for the patients who were not diagnosed with a structured diagnostic interview. This limitation is mitigated by the observation that the results of the subgroup analysis on SCID-diagnosed patients were very similar to those of the full sample.

Given these limitations, some measure of caution is warranted in interpreting the findings of this study, which await replication and extension. Studies using assessment instruments that are more suitable than the BPRS to assess delusions would be particularly valuable.

The present study contributes to the line of research on the relationship between delusional themes and psychiatric diagnosis. It can only partially address this issue due to the absence of data about delusions other than the four types specifically covered by the BPRS. Overall, our findings appear to suggest a middle position in the debate between scholars from a cognitive approach and scholars following a classical psychopathological approach.

On the one hand, the close link between grandiose delusions and mania, and the even closer link between delusions of guilt and depression, suggest that these types of delusion may differ from other kinds of delusion. A phenomenological analysis of the delusional experience may give back meaning to the arising of such delusions in the context of distinct clinical entities, as is the case with the guilt paradigm. In this respect, a careful examination of delusional themes may be a helpful tool for the clinician in the diagnostic process and, therefore, in the choice of treatment. The phenomenological approach may represent a valuable framework for gathering information to make sense of the delusional experience which, in isolation from its psychopathological context, has limited diagnostic value (20). It should nevertheless be noted that although delusions of grandiosity and guilt were found to characterize manic and depressive episodes, respectively, their differential diagnostic value is limited by the fact that only a minority of the patients with a psychotic mood disorder experienced such delusions. The differential diagnostic value of delusion is further limited by the fact that in a substantial proportion of patients the clinical presentation is primarily characterized by psychotic symptoms other than delusion, and that some psychotic patients do not show any type of delusion.

On the other hand, the widespread observation of persecutory and somatic delusions suggests the usefulness of searching for non-specific pathogenic mechanisms. Indeed, for a number of seemingly ubiquitous delusions, the research work aimed at identifying shared patterns and etiologies that are being carried out within neurocognitive science has potential to lead to significant therapeutic advances. Future studies may fruitfully integrate these approaches by examining both the content of delusions and their relation to the cognitive, perceptual, and emotional aspects of information processing.

## Ethics Statement

According to the Italian legislation, this study does not need formal ethical approval because it is a retrospective, purely observational study based on data collected as part of routine patient assessment.

## Author Contributions

AP designed the study, contributed to the coordination and supervision of data collection, performed data quality control and statistical analysis, and drafted the manuscript. LF contributed to study conception and to coordination of data collection, contributed to literature review, and revised the manuscript for important intellectual content. MP contributed to study conception and to coordination of data collection, contributed to literature review, and revised the manuscript for important intellectual content. AG contributed to coordination and supervision of data collection and revised the manuscript for important intellectual content. MB provided inputs for study design, contributed to coordination and supervision of data collection, and revised the manuscript for important intellectual content. All authors contributed to and approved the final manuscript, and agreed to be accountable for all aspects of the work.

## Conflict of Interest Statement

The authors declare that the research was conducted in the absence of any commercial or financial relationships that could be construed as a potential conflict of interest.
