# Supplementary material for: Delusional Themes Across Affective and Non-Affective Psychoses
Source: Front Psychiatry. 2018 Apr 5;9:132. doi: 10.3389/fpsyt.2018.00132 (PMC5895977; doi:10.3389/fpsyt.2018.00132)
Supplement: Supplementary file 1 [file table_1.PDF]

Supplementary Table 1. Presence of specific delusional themes by diagnostic group in the full sample (N=830): observed frequencies and adjusted standardised residuals

	**Delusions of guilt [N (Res)]**	**Delusions of grandiosity [N (Res)]**	**Persecutory delusions [N (Res)]**	**Somatic delusions [N (Res)]**
**Schizophrenia (N=318)**	3 (-5.0)	22 (-1.3)	88 (3.2)	24 (-0.1)
**Delusional disorder (N=95)**	0 (-2.7)	6 (-0.8)	43 (5.9)	12 (1.9)
**Schizoaffective disorder, manic type (N=29)**	0 (-1.4)	3 (0.4)	9 (1.2)	1 (-0.9)
**Schizoaffective disorder, mixed type (N=29)**	2 (0.1)	1 (-1.0)	5 (-0.6)	7 (3.4)
**Schizoaffective disorder, depressive type (N=60)**	5 (0.7)	0 (-2.5)	7 (-2.0)	2 (-1.3)
**Bipolar I disorder, current episode manic (N=162)**	0 (-3.7)	33 (6.0)	13 (-4.7)	2 (-3.4)
**Bipolar I disorder, current episode mixed (N=32)**	2 (0.0)	6 (2.1)	5 (-0.9)	4 (1.0)
**Bipolar disorder, current episode depressed with psychotic features (N=23)**	7 (4.9)	0 (-1.5)	6 (0.5)	4 (1.8)
**Major depressive disorder with psychotic features (N=82)**	33 (13.4)	0 (-2.9)	5 (-3.6)	8 (0.7)

N= observed frequency; Res = Adjusted standardised residual
