# Supplementary material for: Delusional Themes Across Affective and Non-Affective Psychoses
Source: Front Psychiatry. 2018 Apr 5;9:132. doi: 10.3389/fpsyt.2018.00132 (PMC5895977; doi:10.3389/fpsyt.2018.00132)
Supplement: Supplementary file 2 [file table_2.PDF]

Supplementary Table 2. Presence of specific delusional themes by diagnostic group in patients diagnosed with the SCID-I (N=348): observed frequencies and adjusted standardised residuals

	Delusions of guilt [N (Res)]	Delusions of grandiosity [N (Res)]	Persecutory delusions [N (Res)]	Somatic delusions [N (Res)]
**Schizophrenia (N=116)**	2 (-3.7)	7 (-0.9)	33 (1.8)	7 (-0.3)
**Delusional disorder (N=42)**	0 (-2.3)	1 (-1.4)	21 (4.5)	3 (0.1)
**Schizoaffective disorder, manic type (N=9)**	0 (-1.0)	2 (1.6)	1 (-0.8)	0 (-0.8)
**Schizoaffective disorder, mixed type (N=7)**	0 (-0.9)	0 (-0.8)	1 (-0.5)	1 (0.8)
**Schizoaffective disorder, depressive type (N=24)**	2 (-0.3)	0 (-1.5)	4 (-0.7)	0 (-1.4)
**Bipolar I disorder, current episode manic (N=74)**	0 (-3.2)	16 (5.0)	7 (-3.1)	1 (-2.1)
**Bipolar I disorder, current episode mixed (N=11)**	1 (-0.1)	1 (0.2)	3 (0.4)	1 (0.3)
**Bipolar disorder, current episode depressed with psychotic features (N=14)**	6 (4.2)	0 (-1.1)	6 (1.8)	4 (3.4)
**Major depressive disorder with psychotic features (N=51)**	24 (9.5)	0 (-2.2)	3 (-3.1)	6 (1.6)

N= observed frequency; Res = Adjusted standardised residual
